# Three-dimensional anatomical reconstruction as a guideline-anchored framework for choosing between lung-sparing resection and image-guided ablation in deep subsolid pulmonary nodules: a thoracic surgeon’s perspective

**DOI:** 10.3389/fonc.2026.1981772

**Published:** 2026-09-28

**Authors:** Zhe Li, Huixian Li

**Affiliations:** Department of Thoracic Surgery, Shijiazhuang People’s Hospital, Shijiazhuang, Hebei, China

**Keywords:** ground-glass nodule, microwave ablation, sublobar resection, subsolid pulmonary nodule, three-dimensional reconstruction, treatment decision-making

## Abstract

Low-dose computed tomography screening has markedly increased the detection of small, deeply located subsolid pulmonary nodules, and local therapy for these lesions now encompasses anatomical segmentectomy, tailored sublobar resection, lobectomy, and image-guided thermal ablation. Although multidisciplinary pathways already inform modality selection, three-dimensional (3D) anatomical reconstruction - despite its established role in segmentectomy planning - is rarely used as a shared patient-specific dataset to guide both resection and ablation decisions. In this perspective, a thoracic surgeon argues that, for a deep or cross-segment subsolid nodule with an established indication for local therapy, a single patient-specific 3D dataset can inform both branches of the decision. The same volumetric anatomy - including nodule size and consolidation-to-tumor ratio, segmental boundaries, bronchovascular relationships, pleura, and the chest wall - is first used to determine whether adequate-margin lung-sparing resection is achievable and, if it is not, to assess whether ablation is anatomically feasible through three sequential filters: trajectory safety, perilesional structural risk, and predicted ablative coverage. The approach is illustrated with a representative case and discussed in the contexts of multifocal disease, the post-resection lung, and perivascular lesions, together with the trade-offs associated with forgoing nodal staging and tissue diagnosis. The framework does not expand indications beyond those already supported by guidelines. Rather, it operationalizes these recommendations through patient-specific anatomy, with the aim of making modality selection more transparent, reproducible, and auditable. It remains an unvalidated, single-center proposal that requires prospective evaluation.

## Introduction

Low-dose computed tomography (LDCT) screening has reshaped the spectrum of pulmonary nodules encountered in thoracic practice. The lesion under consideration is increasingly small, peripheral or deeply intraparenchymal, frequently subsolid, and sometimes one of several synchronous nodules in the same patient (13, 14). During the same period, thoracic surgery has moved from lobectomy as a near-universal standard toward parenchyma-sparing procedures - including anatomical segmentectomy, combined subsegmentectomy, and extended segmentectomy - tailored to lesion size, location, and radiological appearance (15). This transition has been reinforced by randomized evidence supporting sublobar resection for selected small peripheral tumors (1, 2). In parallel, image-guided thermal ablation, particularly microwave ablation (MWA), has emerged as a lung-preserving option for patients who are medically inoperable, have limited pulmonary reserve or multifocal disease, or decline surgery (5), although the evidence base for ablation in deep and subsolid lesions remains less mature than that for resection (16).

For many indolent pure ground-glass lesions, observation remains appropriate, and overtreatment is a genuine concern. The framework discussed here applies only after a lesion has a recognized indication for local therapy. Once intervention is warranted, the practical question for a deep subsolid nodule is often not whether to operate, but how to choose defensibly among several lung-sparing options and ablation. We contend that this choice is fundamentally anatomical and that the same 3D reconstruction increasingly used for segmentectomy planning can - and should - inform the entire local-treatment decision. Throughout this article, we use a shortest lesion-to-pleura distance greater than approximately 2 cm as the primary operational definition of a deep lesion, with location in the inner two-thirds of the lung serving as a supportive descriptor; when these two descriptors disagree, the pleural-distance criterion takes precedence. The aim is not to adjudicate a contest between resection and ablation, but to describe how a guideline-anchored 3D framework can make selection more transparent, reproducible, and auditable, while explicitly identifying where the approach rests on expert opinion rather than validated evidence.

## Guideline anchors and the dual role of one 3D dataset

The framework introduces no indication beyond those already endorsed; each step represents an operational refinement of existing recommendations. Surgically, JCOG0802 and CALGB/Alliance 140503 established segmentectomy and sublobar resection, respectively, as non-inferior approaches for peripheral tumors no larger than 2 cm (1, 2), and JCOG0804 showed that ground-glass opacity (GGO)-predominant lesions with a consolidation-to-tumor ratio (CTR) of 0.25 or less can be managed by sublobar - predominantly wedge - resection (3). Contemporary consensus frames the extent of resection by lesion size, CTR, location, and the feasibility of achieving an adequate margin (4). As a pragmatic stratification, we treat CTR <=0.25 as low (GGO-predominant), 0.25-0.5 as intermediate, and >0.5 as solid-predominant. An adequate margin is not a single fixed value: for solid-predominant lesions, a parenchymal margin of at least 2 cm or the tumor diameter, whichever is greater, is commonly applied (4). For very low-CTR peripheral lesions, less extensive resection may be appropriate when a sufficient margin is secured; in JCOG0804, intraoperative confirmation of a minimum 5-mm margin was required, with conversion to a more extensive resection when the margin was insufficient (3). In our practice, 3D reconstruction is used preoperatively to estimate the spatial relationship between the lesion and the proposed transection plane and to estimate the expected parenchymal margin, rather than to replace intraoperative or pathological margin assessment. During surgery, margin adequacy is judged by integrating lesion localization, inspection and palpation of the resected specimen when feasible, and pathological assessment when clinically indicated. Thus, a 3D-derived margin is a planning estimate; the final surgical margin is reported according to the operative and pathology records. When the pathology report documents only a negative margin or R0 resection without a measured distance, we report the margin as negative/R0 and do not infer a numerical value. For ablation, society and expert consensus statements support its use in medically inoperable patients, multifocal disease, the residual lung, and clinical situations in which lung preservation is prioritized (5, 19). A recent AATS consensus further identifies sublobar resection, stereotactic body radiotherapy (SBRT; also termed stereotactic ablative radiotherapy [SABR]), and thermal ablation as applicable options for high-risk stage I disease (24).

The value of 3D reconstruction in surgery is well established. It clarifies the relationship of the lesion to the segmental and subsegmental bronchi, pulmonary arteries, and pulmonary veins; supports segmentectomy planning; and allows the expected margin to be assessed in three dimensions rather than inferred from axial images (6–8). Recent comprehensive reviews have focused predominantly on these surgical applications and, increasingly, on artificial-intelligence-driven modeling (12, 21, 22). By contrast, the application of the same reconstruction to the ablation decision has received comparatively little attention. Yet the identical dataset contains much of the information required for ablation assessment. A trajectory-oriented review of the same model can support assessment of whether a safe percutaneous route exists, whether the ribs or scapula obstruct it, and how the proposed path relates to critical thoracic structures (17). Proximity to pulmonary vessels and bronchi is then considered separately because it may influence heat-sink and injury risk during thermal ablation (5). The feasibility of circumferential ablative coverage is also anatomical, and a single-center study that quantified the minimal ablative margin after MWA linked a larger margin to improved local control (9). The central but underused observation is therefore that the same 3D model that defines resection feasibility can also define ablation feasibility, with both assessments drawing on overlapping anatomy interpreted through different therapeutic questions.

## A unified, guideline-anchored 3D framework

After 3D reconstruction, the decision proceeds through sequential nodes (**Figure 1**). Node A asks whether an adequate margin can be achieved within a single anatomical segment; if so, anatomical segmentectomy is reasonable. If the lesion crosses a segmental plane, Node B asks whether a tailored resection - combined subsegmentectomy or extended segmentectomy - can reconcile an adequate margin with meaningful parenchymal preservation. Cross-segment location is therefore not, by itself, an indication for ablation. When parenchyma-sparing resection can no longer reconcile margin with preservation, Node C uses multidisciplinary, patient-factor-weighted selection among lobectomy, SBRT, and ablation evaluation, according to cardiopulmonary reserve, oncological risk and nodal staging requirements, frailty and comorbidity, multifocal disease, prior resection, future pulmonary reserve, and patient values and preference. Patients who can tolerate definitive resection and for whom oncological radicality and pathological nodal staging are prioritized proceed to lobectomy; SBRT represents a parallel non-surgical option when surgery is unsuitable or a safe percutaneous route is unavailable; and patients in whom lung preservation, frailty/comorbidity, prior resection, or multifocal disease may favor image-guided thermal ablation proceed to explicit ablation-feasibility assessment. A decision to ablate, like wedge resection, entails forgoing systematic nodal dissection. In a National Cancer Database analysis of patients with 1.5- to 2.0-cm clinical stage IA2 NSCLC, no lymph nodes were sampled in 21.7% of patients undergoing sublobar resection compared with 2.1% undergoing lobectomy (28). Ablation and radiotherapy also do not provide surgical nodal staging, which complicates cross-modality survival comparisons and may contribute to apparent outcome differences (24–26). When the probability of nodal involvement is not negligible, anatomical resection may remain preferable despite its greater parenchymal cost.

**Figure 1:**
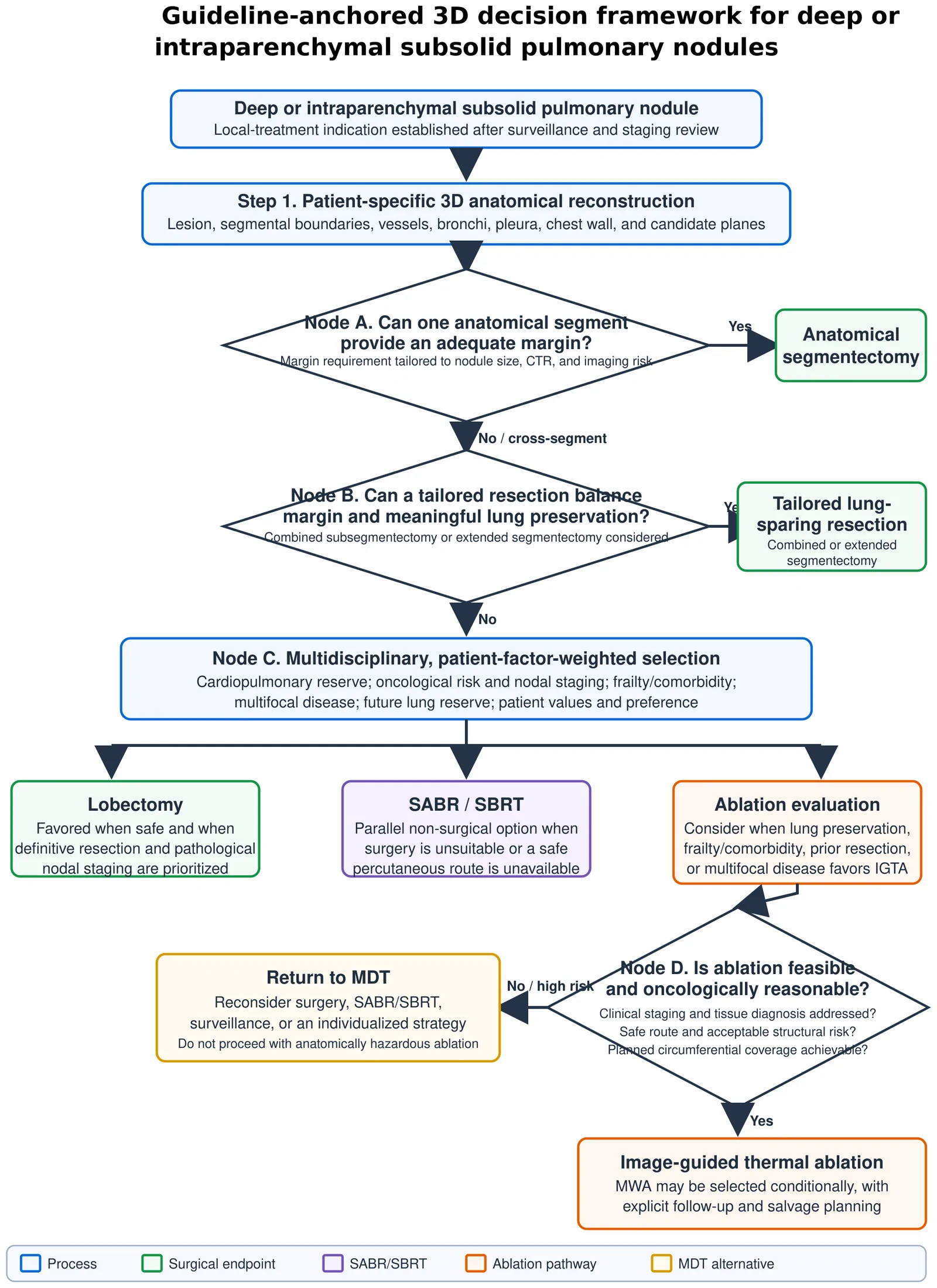
Guideline-anchored 3D decision framework for deep or intraparenchymal subsolid pulmonary nodules. After a local-treatment indication has been established, one patient-specific 3D anatomical reconstruction is used to assess lesion location, segmental boundaries, vessels, bronchi, pleura, chest wall, and candidate resection planes. Node A assesses whether one anatomical segment can provide an adequate margin; Node B considers whether combined subsegmentectomy or extended segmentectomy can balance margin adequacy with meaningful lung preservation; Node C provides multidisciplinary, patient-factor-weighted selection among lobectomy, SABR/SBRT, and ablation evaluation, incorporating future pulmonary reserve among the patient-level considerations; and Node D determines whether ablation is anatomically feasible and oncologically reasonable on the basis of staging and tissue diagnosis, route and structural risk, and planned circumferential coverage. Patients with high-risk or unsuitable ablation anatomy return to MDT review, whereas patients proceeding to ablation require explicit follow-up and salvage planning. CTR, consolidation-to-tumor ratio; MDT, multidisciplinary team; MWA, microwave ablation; SABR, stereotactic ablative radiotherapy; SBRT, stereotactic body radiotherapy; IGTA, image-guided thermal ablation.

Node D then tests whether ablation is anatomically feasible, not merely whether the lesion can be reached. We focus on MWA because, compared with radiofrequency ablation, it is less affected by tissue impedance and charring and can achieve higher intratumoral temperatures (16); its clinical use in peripheral GGO-predominant lung adenocarcinoma has also been reported (18). The same anatomical reasoning also applies conceptually to cryoablation. Feasibility is assessed through three filters. The first, trajectory safety, requires a route that avoids vessels and their larger branches, bronchi, fissures, adhesions and bullae, and bony or mediastinal obstruction. As a pragmatic institutional threshold, an intrapulmonary path longer than approximately 5–7 cm is considered to increase technical difficulty and prompts reassessment. The second, perilesional structural risk, gives particular attention to vessels at least 3 mm in diameter. Within our framework, a lesion-to-vessel distance below 5 mm is considered high risk for heat-sink and incomplete ablation, 5–10 mm intermediate risk, and greater than 10 mm relatively safe. The path-length threshold, vessel-caliber threshold, and distance bands reflect institutional practice and are proposed as pragmatic starting points rather than validated cut-offs. The third filter, predicted ablative coverage, verifies that the anticipated ablation zone encompasses the lesion with a margin. When anatomy permits, we target a minimal ablative margin of at least 5 mm beyond the visible boundary (9), using overlapping ablations when one aspect of the margin - typically the vessel-facing side - would otherwise remain insufficiently covered. A separate issue at this node is tissue diagnosis: because ablation yields no surgical specimen, we obtain histology whenever feasible, either by synchronous or prior biopsy, reserve empirical ablation for specifically defined circumstances, and acknowledge the absence of pathological confirmation in the multidisciplinary record. When these conditions cannot be met, the patient proceeds to an MDT-based alternative, including surgery, SBRT, or surveillance. When ablation is selected, explicit imaging follow-up and salvage planning are incorporated into the treatment strategy.

At Node D, the unifying claim becomes concrete: the dataset that quantified the resection margin now quantifies ablation feasibility. **Figure 2** illustrates this concept in a representative ablation case in which reconstruction showed that adequate lung-sparing resection would have required excessive parenchymal sacrifice. When the framework was applied to the single dataset, no sublobar plane could achieve an adequate margin without disproportionate loss, arguing against resection. On the ablation side, the planned intrapulmonary path was approximately 7 cm - at the upper limit that our framework regards as more technically demanding but acceptable after trajectory review - the lesion lay approximately 6 mm from the nearest vessel of at least 3 mm (intermediate risk by our criteria), and a predicted ablative margin of approximately 10 mm could be achieved. The planned percutaneous trajectory matched the intraprocedural antenna position, and the procedure was completed uneventfully, without pneumothorax or significant hemorrhage. The same model thus argued against adequate resection while supporting ablation feasibility.

**Figure 2:**
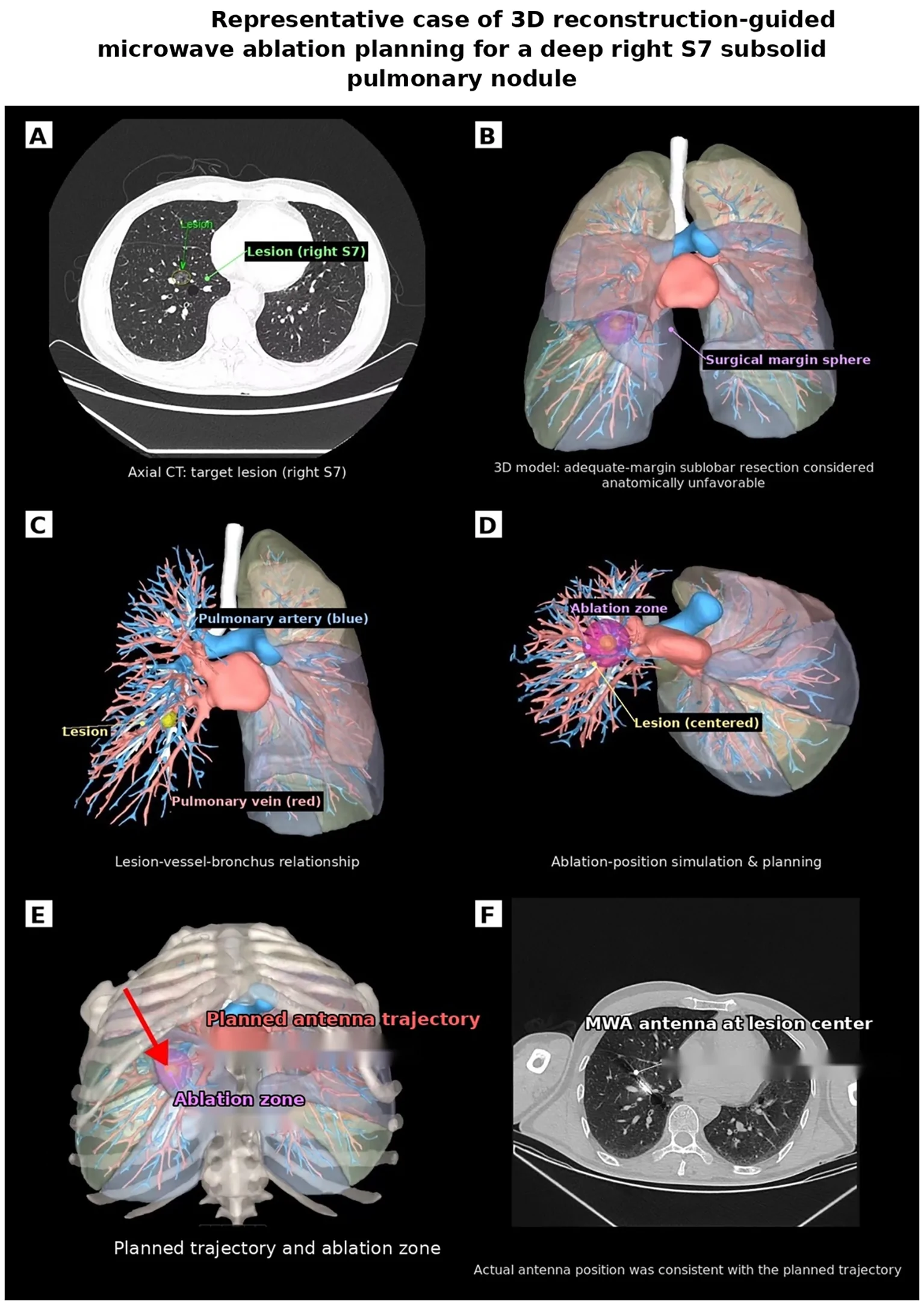
Representative case of 3D reconstruction-guided microwave ablation planning for a deep right S7 subsolid pulmonary nodule. This representative ablation case is separate from the multifocal case described in the text. **(A)** Axial CT shows the target lesion in the right S7 segment. **(B)** Patient-specific 3D reconstruction with a surgical margin sphere shows that adequate-margin sublobar resection is anatomically unfavorable. **(C)** Three-dimensional reconstruction demonstrates the lesion-vessel-bronchus relationship; pulmonary arteries are shown in blue and pulmonary veins in red. **(D)** Ablation-position simulation centers the lesion within the planned ablation zone. **(E)** Three-dimensional planning depicts the intended antenna trajectory and ablation zone. **(F)** Intraprocedural axial CT confirms the MWA antenna at the lesion center, with the actual antenna position consistent with the planned trajectory. In this case, the planned intrapulmonary path was approximately 7 cm, the lesion-to-nearest-vessel distance for a vessel at least 3 mm in diameter was approximately 6 mm, and the predicted ablative margin was approximately 10 mm. MWA, microwave ablation.

## Application to special scenarios

The deep intraparenchymal nodule is the prototype case, because no modality is straightforward when a lesion is both deep and subsolid. In multifocal disease, the goal shifts toward stewardship of total lung reserve. 3D reconstruction can help prioritize resection of a dominant lesion - defined by integrating solid-component size, CTR, growth, and morphological suspicion rather than size alone - while assigning secondary lesions to ablation when the three filters are satisfied or to surveillance when they are low-risk and stable (4, 23). In a separate representative patient with multiple right-lung nodules, combined subsegmentectomy of the right lower lobe yielded a moderately to well differentiated adenocarcinoma (2.5 x 2.5 x 1.4 cm) with negative nodal sampling across all examined stations (0/11). When middle- and upper-lobe nodules subsequently enlarged, reconstruction allocated treatment according to anatomy. The deeper pure ground-glass middle-lobe lesion was treated by MWA because a safe trajectory and circumferential coverage were achievable. No pre-ablation biopsy was performed for this lesion because its deep location increased the procedural burden and risk of an additional puncture, while pathological confirmation was not expected to change the planned local treatment; the absence of tissue confirmation was therefore accepted as a limitation of this lesion-specific management decision. The more peripheral upper-lobe lesion was treated by thoracoscopic wedge resection in the same session. For surgically resected lesions, we report the documented pathological margin status; when the pathology report records only a negative margin/R0 resection without a numerical distance, no millimeter value is inferred. At 2 months, the ablation zone was satisfactory with progressive shrinkage, reflecting an early local response rather than a definitive oncological endpoint. In the residual lung after prior resection, where repeat surgery may exact a disproportionate cost, reconstruction of the distorted post-surgical anatomy supports both the feasibility of further resection and the safety of an ablation trajectory (20). For nodules abutting a vessel or bronchus, the same perilesional relationship simultaneously informs the difficulty of surgical dissection and the heat-sink and injury risks of ablation.

Any framework that weighs ablation against resection must also consider SBRT, a well-established non-invasive option for medically inoperable early-stage disease with a more mature evidence base than ablation in that setting. A companion AATS systematic review found that, in the absence of completed randomized trials, sublobar resection generally provided superior local control and overall survival compared with ablation, whereas ablation and SBRT were broadly comparable in selected patients (24, 25). A recent meta-analysis was consistent with this hierarchy, reporting worse overall survival with SBRT than with sublobar resection (hazard ratio 1.51, 95% CI 1.18-1.93) and pooled 3-year overall survival of approximately 80% versus 64% for SBRT and ablation; these differences were attributed in part to the poorer baseline health of non-surgical candidates (26). Within our framework, SBRT is therefore a parallel option that the same anatomical assessment can help select: when a percutaneous route is unsafe or perivascular risk is prohibitive, the model redirects the discussion toward SBRT or surgery rather than hazardous ablation.

## Discussion

At present, selection of local treatment for a deep subsolid nodule may depend as much on which specialist first evaluates the patient as on the anatomy itself, particularly when no shared anatomical basis for decision-making is used. This is the gap that the present framework seeks to address. The claim of this perspective is deliberately modest: not that any one modality is superior, nor that a unified approach improves outcomes, but that anchoring the resection-versus-ablation decision in one shared anatomical dataset may make it more consistent, more complete, and more auditable. Its rationale concerns decision quality rather than proven efficacy. Interpreting the same dataset for both modalities may promote consistency, so that a deep nodule is less likely to be routed differently simply because it is first assessed by a surgeon rather than an interventional radiologist - although we have no quantitative data on how often such divergence occurs, and this remains a hypothesis to be tested. It may promote completeness by reducing the chance that resectability is judged without considering ablation feasibility, or vice versa. It may also promote auditability by creating an explicit anatomical record of why a modality was chosen. This anatomical, pretreatment approach is complementary to work using post-treatment outcome prediction - including a deep-learning model based on pretreatment CT that identifies patients suited to ablation (27) - which predicts outcomes rather than explaining an anatomical decision. The two approaches address the same clinical selection problem from different directions and may ultimately be combined.

Several limitations define the boundaries of this proposal. Most fundamentally, it is an unvalidated, single-center expert framework. Its central claims have not been tested prospectively, and the specific thresholds we apply - an intrapulmonary path of approximately 5–7 cm, lesion-to-vessel distance bands, and a minimal ablative-margin target - are pragmatic and hypothesis-generating rather than evidence-derived; they are intended to make our reasoning explicit and reproducible. Long-term oncological evidence for ablation of deep subsolid nodules remains limited, and some series report poorer local control after ablation than after sublobar resection despite comparable survival (10). Much of the favorable ablation experience also concerns indolent disease, so reported outcomes may partly reflect case selection. The quality of reconstruction varies with software, segmentation method, CT acquisition quality, and operator input, all of which can affect the margin and distance measurements on which the framework depends (11, 22). In addition, preprocedural models are static representations of a dynamic situation (6). Post-ablation imaging can also be difficult because reactive hyperemia, inflammation, and other treatment-related changes may complicate delineation of the true ablation zone (9). For subsolid lesions specifically, standardized imaging criteria remain an unmet need. Finally, generating these models adds time and depends on software and expertise that may not be uniformly available, which may limit reproducibility in resource-constrained settings. The framework should therefore be understood as a structured way to organize an anatomical decision within the current evidence base, not as a substitute for the randomized data still needed. A concrete next step would be a prospective registry in which deep subsolid nodules are independently assessed with and without the shared 3D framework, comparing the consistency of modality recommendations between assessors and linking 3D-derived parameters - including margin, trajectory length, lesion-to-vessel distance, and predicted ablative margin - to procedural and oncological outcomes. Such studies are required before the approach can be recommended beyond the experience that motivated it.

## Data Availability

The original contributions presented in the study are included in the article/supplementary material. Further inquiries can be directed to the corresponding author.
